# Potential roles of the rectum keystone microbiota in modulating the microbial community and growth performance in goat model

**DOI:** 10.1186/s40104-023-00850-3

**Published:** 2023-04-07

**Authors:** Dangdang Wang, Guangfu Tang, Lichao Zhao, Mengya Wang, Luyu Chen, Congcong Zhao, Ziqi Liang, Jie Chen, Yangchun Cao, Junhu Yao

**Affiliations:** College of Animal Science and Technology, Northwest A&F University, Yangling, 712100 Shaanxi China

**Keywords:** Early life, Goat, Growth performance, Rectum microbiota, SCFA

## Abstract

**Background:**

Ruminal microbiota in early life plays critical roles in the life-time health and productivity of ruminant animals. However, understanding of the relationship between gut microbiota and ruminant phenotypes is very limited. Here, the relationship between the rectum microbiota, their primary metabolites, and growth rate of a total of 76 young dairy goats (6-month-old) were analyzed, and then 10 goats with the highest or lowest growth rates respectively were further compared for the differences in the rectum microbiota, metabolites, and animal’s immune parameters, to investigate the potential mechanisms by which the rectum microbiota contributes to the health and growth rate.

**Results:**

The analysis of Spearman correlation and microbial co-occurrence network indicated that some keystone rectum microbiota, including unclassified Prevotellaceae, *Faecalibacterium* and *Succinivibrio*, were the key modulators to shape the rectum microbiota and closely correlated with the rectum SCFA production and serum IgG, which contribute to the health and growth rate of young goats. In addition, random forest machine learning analysis suggested that six bacterial taxa in feces could be used as potential biomarkers for differentiating high or low growth rate goats, with 98.3% accuracy of prediction. Moreover, the rectum microbiota played more important roles in gut fermentation in early life (6-month-old) than in adulthood stage (19-month-old) of goats.

**Conclusion:**

We concluded that the rectum microbiota was associated with the health and growth rate of young goats, and can be a focus on the design of the early-life gut microbial intervention.

**Supplementary Information:**

The online version contains supplementary material available at 10.1186/s40104-023-00850-3.

## Background

Domestic ruminants are characterized by a complex gastrointestinal system, which can convert low-quality dietary substrates that are unsuitable for human consumption into animal protein. Typically, scientists have focused much of their attention on understanding the ruminal micro-ecosystem and revealed that rumen microbiome features can be linked to the animal’s economically important traits, such as feed digestion efficiency [1, 2], and milk production [3]. However, microbiome in the hindgut also plays an important role in ruminant nutrition, health, and productivity, particularly in early life of animals [4, 5], hence, work in this area needs to be further investigated.

In humans and mice, recent studies have demonstrated the existence of the mechanistic causal relationship between gut microbiota and host health [6–8]. More and more studies are investigating the importance of hindgut microbiota in the health and production of livestock [9]. Several systematic studies revealed the correlation of hindgut microbiome with pig phenotypes, and found that fecal microbiota transplantation can introduce some specific bacteria to the recipients, which can prevent animal diarrhea [10], and promote host fat accumulation [11] and growth performance [12]. In ruminants, there are a few studies that explored the hindgut microbial composition and function, compared with the studies conducted in the rumen [13]. These studies suggested a link between hindgut microbiota and ruminant production and health [14, 15]. Furthermore, hindgut microbial fermentation of carbohydrates produces a wide-array of microbial metabolites (such as short-chain fatty acids, SCFA), which are beneficial to the host and bacterial growth [13, 16], and play a role in regulating the immune system and inflammatory responses [17, 18]. However, our understanding of how the hindgut microbiota and their metabolites affect the health and growth performance of ruminant animals is limited, and the best time window for modulation of hindgut microbiota remain unclear.

Therefore, we raised two questions in the present study: (1) How does the hindgut microbiota affect the growth performance and health of young ruminants? and (2) Could the hindgut microbiota and their metabolites be used as markers for classifying the young animals with different growth rates? To answer the questions, we used 76 young goats with different growth rates as model animals, fed them with the same diet, kept them under the same condition, determined their rectum microbiota in young and adult stages, and analyzed the relationships between the animal’s growth rate and microbiota features or metabolites. These studies may help us to develop potent community modulation and intervention strategies for improving feed efficiency and growth performance of young ruminants.

## Materials and methods

### Ethics statement

This experiment was conducted at the Animal Research and Technology Center of Northwest A&F University (Yangling, Shaanxi, China, 34.2722° N, 108.0846° E), and it was performed in accordance with the recommended guidelines from the Administration of Affair Concerning Experimental Animals (Ministry of Science and Technology, China, revised 2004). The protocol was approved by the Institutional Animal Care and Use Committee at Northwest A&F University.

### Experiment animals and sampling

A total of 76 healthy, female, Guanzhong goats were initially used in this experiment. They had no history of administration of any antimicrobial agents (antibiotics, antifungals or antivirals) or suffering from any infectious disease. Their birth weights were recorded immediately after birth, and kids were reared together under the same environmental condition. After weaning (3-month-old), they were fed the same diet. The diet ingredients and feeding programs are shown in Additional file 1: Table S1.

Goats were weighed at the age of 6-month-old (188.2 ± 0.40 d, mean ± SE) to calculate the average of daily weight gain (ADG, g/d) from birth. The average of ADG for all 76 goats was 109 ± 1.72 g/d with a coefficient of variation of 13.73%. All 76 goats were ranked according their individual ADG, then the top 10 goats were selected as the high growth rate group (HADG, ADG: 132.8 ± 1.86 g/d), and the bottom 10 goats selected as low growth rate group (LADG, ADG: 84.8 ± 2.19 g/d). At the age of 19 months, four goats from both HADG and LADG groups were excluded because of diarrhea [two goats], fever [one goat], or dermatoses [one goat], so there were 8 adult goats in each group for further study, the remained adult HADG and LADG goats were renamed as HAL and LAL (*n* = 8 each group).

Fecal samples of all young goats (6-month-old, *n* = 76), and 16 healthy adult goats (19-month-old, HAL and LAL, *n* = 8 each group) were taken from the rectum. In brief, all sample (76 young goats and 16 adult goats) collection was programmed over 3 d in 3 h intervals so that all 24 samples represented every hour of a 24-h feeding cycle. Samples were immediately snap-frozen in liquid nitrogen. At the end of sampling period, all 24 samples for each goat were pooled, mixed, and homogenized using a sterile slap homogenizer. Then, about 1 g of subsample was taken and stored at −80 °C for metagenomic DNA extraction. All fecal samples were subjected to fecal dry matter determination [19]. The remainder sample was stored at −20 °C for SCFA extraction and analysis.

Blood samples were drawn from the jugular vein of HADG and LADG goats (*n* = 10 each group) into endotoxin free evacuated tubes 0–1 h before morning feeding. Samples were centrifuged at 3500 × *g* for 15 min at 4 °C for serum collection. All serum samples were stored at −80 °C until further processing and analysis.

The feed intake of HADG and LADG goats (*n* = 10 each group) were measured one week before sampling (171.2 ± 0.40 d). In brief, feed offered to and refused by each goat was recorded continuously for 7 d. The feed samples were dried at 65 °C for 48 h to obtain dry matter content of the ration. Daily dry matter intake (DMI) per goat was calculated by multiplying daily as-fed intake by the dry matter content of the ration. There was no significant difference in DMI between HADG and LADG (1.03 ± 0.03 vs. 1.01 ± 0.02, *P* = 0.735, *t*-test).

### DNA extraction and 16S rRNA gene sequencing

DNA of fecal samples from 76 young goats and 16 adult goats (HAL and LAL) was extracted using the E.Z.N.A.®Stool DNA kit (Omega Bio-Tek, Norcross, GA, USA) according to the manufacturer’s protocol. The DNA concentration was measured with a Nanodrop-2000 (Thermo Fisher Scientific, Wilmington, DE, USA) and the quality was assessed using 1% agarose gel electrophoresis. Bacterial 16S rRNA gene fragments (V3-V4) in the extracted DNA were amplified using the forward primers 338F (5′-ACTCCTACGGGAGGCAGCAG-3′) and the reverse primer 806R (5′-GGACTACHVGGGTWTCTAAT-3′). PCR products were visualized on 2% agarose gels and purified using the QIAquick gel extraction kid (Qiagen, Dusseldorf, Germany). All amplicons were sequenced using the paired-end (PE300) method on a MiSeq platform (Illumina, San Diego, USA) following the standard protocols.

### Illumina sequencing data analysis

The raw sequences were merged with FLASH (v1.2.11) [20] and the quality filtered with fastp (0.19.6) [21]. Sequences were imported into QIIME2 v2021.8 for demultiplexing and the construction of an amplicon sequence variant (ASV) table using DADA2 [22]. Bacterial 16S ASVs were assigned a taxonomy using the SILVA database (version 138) as the reference, singletons were removed and a table of ASV counts per sample was generated. Furthermore, phylogenetic investigation of communities by reconstruction of unobserved states 2 (PICRUSt2) analysis (https://github.com/picrust/picrust2) [23] was used to predict the metagenome based on the ASV table, and then the metagenome functions were predicted and the data were exported into levels 1 and 2 of Kyoto Encyclopedia of Genes and Genomes (KEGG) database pathways.

Alpha diversity indices including the richness estimate and Shannon diversity index were calculated using QIIME 2 at the ASV level. The principal coordinate analysis (PCoA) was performed based on Bray-Curtis distance, and statistical significance was determined using analysis of similarities (ANOSIM) with 999 permutations at the ASV level.

### Fecal SCFA, ammonia nitrogen and lactate assays

The concentrations of SCFA (acetate, propionate, butyrate, valerate, isobutyrate, isovalerate and 2-methylbutyrate) were determined using gas chromatography (Agilent 7820A, Santa Clara, CA, USA) with a capillary column (AE-FFAP of 30 m × 0.25 mm × 0.33 μm; ATECH Technologies Co., Lanzhou, China) according to method described by a previous study [24]. In brief, the fecal samples were weighed (about 1.0 g) and added to 3.0 mL distilled water and vigorously vortexed, and the mixture was centrifuged at 12,000 × *g* at 4 °C for 15 min for ammonia nitrogen, lactate, and SCFA analyses. To take into account the variation in water content between fecal samples, the final concentrations (ammonia nitrogen, lactate, and SCFA) were adjusted by fecal dry matter.

For determination of SCFA, 2 mL supernatant was mixed with 400 μL of 25% metaphosphoric acid (w/v), after standing for 4 h at 4 °C, the mixture was centrifuged at 16,000 × *g* at 4 °C for 10 min. Then 200 μL crotonic acid (10 g/L) was added to an aliquot (200 μL) of the supernatant and then filtered through a 0.45 μm filter. The injector and detector temperatures were set at 200 and 250 °C, respectively. The column temperature was increased from 45 °C to 150 °C at a 20 °C/min ramp and then held for 5 min.

The ammonia nitrogen concentration in feces was determined using the colorimetric method of Broderick et al. [25]. And the lactate concentration in the supernatant was assayed by spectrophotometry using a commercial kit (Nanjing Jiancheng Co., Nanjing, China).

### Detection of serum immune parameters

The serum IgG concentration was determined using a goat IgG ELISA Quantitation set (Bethyl Laboratories, Montgomery, TX, USA) according to the manufacturer’s protocol. Serum was diluted in Tris-buffered saline (TBS)-Tween 20 (50 mmol/L Tris, 0.14 mol/L sodium chloride, 0.05% Tween 20) to a final dilution factor of 2.5 × 10^5^. Absorbance was read using a BioTek Synergy HT micro plate reader (BioTek Instruments, Inc., Winooski, VT, USA) at a wavelength of 450 nm. The serum albumin (ALB) and globulin (GLB) were assayed as described by Fan et al. [26]. The AGR was calculated as ALB divided by GLB.

### Construction of microbial co-occurrence networks based on random matrix theory

The global microbial co-occurrence network was constructed for the rectum microbial community in HADG and LADG goats using a random matrix theory (RMT) based pipeline with default parameters as described by Deng et al. [27] to identify microbial interactions. Briefly, The ASVs detected in < 50% of all samples were excluded due to a drastic effect of ASV sparsity on the precision and sensitivity of network inference [27, 28]. A similarity matrix, which measures the degree of concordance between the abundance profiles of individual ASVs across different samples, was then obtained by using Pearson correlation analysis of the abundance data [29]. The fast-greedy modularity optimization procedure was used for module separation. The within-module degree (Zi) and among-module connectivity (Pi) were calculated and plotted to generate a scatter plot for each network. In this study, we used the simplified classification as follows: (i) Peripheral nodes (Zi ≤ 2.5, Pi ≤0.62), which had only a few links and almost always to the nodes within their modules, (ii) Connectors (Zi ≤ 2.5, Pi > 0.62), which were highly linked to several modules, (iii) Module hubs (Zi > 2.5, Pi ≤0.62), which were highly connected to many nodes in their own modules, and (iv) Network hubs (Zi > 2.5, Pi > 0.62), which acted as both module hubs and connectors. From an ecological perspective, peripheral nodes represent specialists whereas the other three are generalists [27].

### Random forest classifier construction

The randomForest package in R was used for the random forest analysis [30] on data of the rectum bacteria, SCFA, and serum immune parameter. For 16S rDNA and SCFA data, each genus and each SCFA were considered as a feature. All the features were taken as training datasets with random forest algorithm using the rfcv function in a R package ‘randomForest’ and then each feature’s importance score was calculated through the permuting values of this feature and then calculating and normalizing the difference of out-of-bag errors before and after a permutation. Meanwhile, mean decrease accuracy (MDA) from the importance matrix was used to select features. Receiver operating characteristic (ROC) analysis was performed to measure the quality of the classification models by the R software package pROC (v1.16.2). ROC curve results were plotted manually by the true positive rate against the false positive rate. ROC curves were constructed, and the area-under-the ROC curve (AUC) was used to designate the ROC effect.

### Statistical analysis

The differences in DMI, fecal SCFA, and serum parameters (ALB, GLB, AGR, and IgG) between two groups (HADG vs. LADG [*n* = 10 each group], HAL vs. LAL [*n* = 8 each group], young goats [6-month-old, *n* = 20] vs. adult goats [19-moth-old, *n* = 16]) were compared using *t* test, and *P* values < 0.05 were considered statistically significant. The differences in rectum microbial α-diversity, family, and genera, KEGG pathways between two groups were compared using Wilcoxon rank-sum test, and *P* values < 0.05 were considered significant. *P* values obtained during multiple comparisons within each analysis were adjusted to reflect the false discovery rate using the Benjamini-Hochberg (HB) algorithm. Correlation analysis between rectum microbiota taxon, serum immune parameters, ADG, and fecal SCFA were performed using Spearman’s rank correlation, and adjusted with the HB false discovery rate. Pairwise correlations (Spearman’s correlation, adjust *P* < 0.05) were used to generate genus-level co-occurrence networks. The module-environmental trait relationships were analyzed using Pearson correlation coefficients. The visualization of the network structure was performed using Cytoscape v3.8.0.

## Results

### Differences in rectum microbiota structure and composition in young goats with different growth rates

**Fig. 1:**
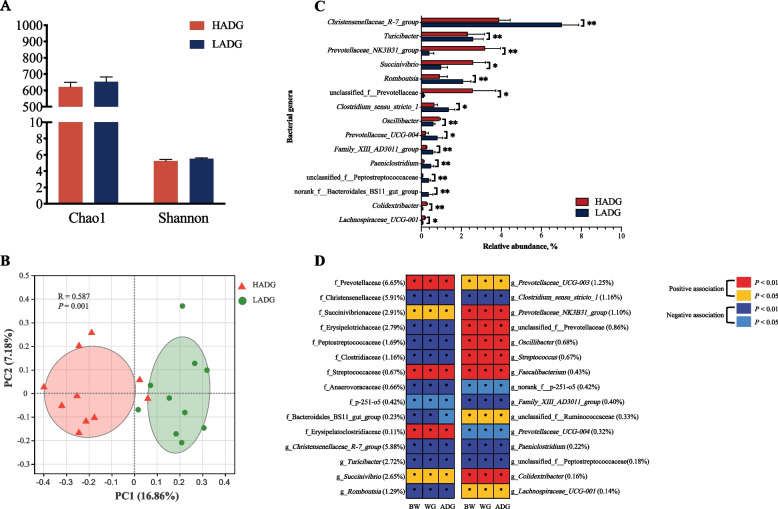
The differences in rectum microbiota diversity and structure between HADG and LADG goats (6-month-old, *n* = 10 each group). **A** The Chao1 and Shannon indexes of rectum microbiota. **B** Principal Coordinate Analysis (PCoA) of rectum microbiota at the ASV level based on the Bray-Curtis dissimilarity. Dissimilarity was analyzed using ANOSIM statistical tests with 999 permutations. **C** Significantly different rectum bacterial genera between HADG and LADG goats. **D** Heatmap showing association between rectum bacterial taxa (average relative abundance > 0.1%) and the growth rate of all young goats (*n* = 76, Spearman’s correlation, *P* < 0.05). The differences in data in (**A**) and (**C**) were tested by the Wilcon rank-sum test. The bars represent mean ± SEM. ^*^P < 0.05, ^**^P < 0.01

There was no significant difference in the rectum microbiota richness and diversity between HADG and LADG young goats (6-month-old, *n* = 10 each group, Fig. 1A). However, PCoA analysis showed differentiation in the rectum bacterial community composition between HADG and LADG goats (*P* = 0.001, Fig. 1B).

Compared to those in LADG goats (Fig. 1C), the abundances of genera *Prevotellaceae NK3B31* group, *Succinivibrio*, unclassified Prevotellaceae, *Oscillibacter*, and *Colidextribacter* were greater in HADG goats (*P* < 0.05). In contrast, genera *Christensenellaceae R-7* group, *Turicibacter*, *Romboutsia*, *Clostridium* sensu stricto 1 and *Prevotellaceae UCG-004* were lesser enriched in HADG goats (*P* < 0.05).

The correlations between the bacterial abundance and the growth rate (from birth to 6-month-old) in all 76 young goats (6-month-old) were analyzed (Fig. 1D, Additional file 1: Table S2). At the family level (relative abundance > 1.0%), the abundances of Prevotellaceae (6.56%) and Succinivibrionaceae (2.91%) were positively correlated with ADG (*P* < 0.05), whereas the abundances of Christensenellaceae (5.91%), Erysipelotrichaceae (2.79%), Peptostreptococcaceae (1.69%), and Clostridiaceae (1.16%) were negatively correlated with ADG (*P* < 0.05). At the genus level (relative abundance > 0.1%), some specific bacterial taxa were positively correlated with the growth rate, such as *Succinivibrio* (2.65%), three members of family Prevotellaceae, *Oscillibacter* (0.68%), *Streptococcus* (0.67%) and *Faecalibacterium* (0.43%), unclassified Ruminococcaceae (0.33%), *Colidextribacter* (0.16%) were positively associated with ADG. The abundance of *Christensenellaceae R-7* group (5.88%), *Turicibacter* (2.72%), *Romboutsia* (1.29%), *Clostridium* sensu stricto 1 (1.16%) were negatively correlated with the growth rate (*P* < 0.05).

### Microbial co-occurrence patterns and network structure

**Fig. 2:**
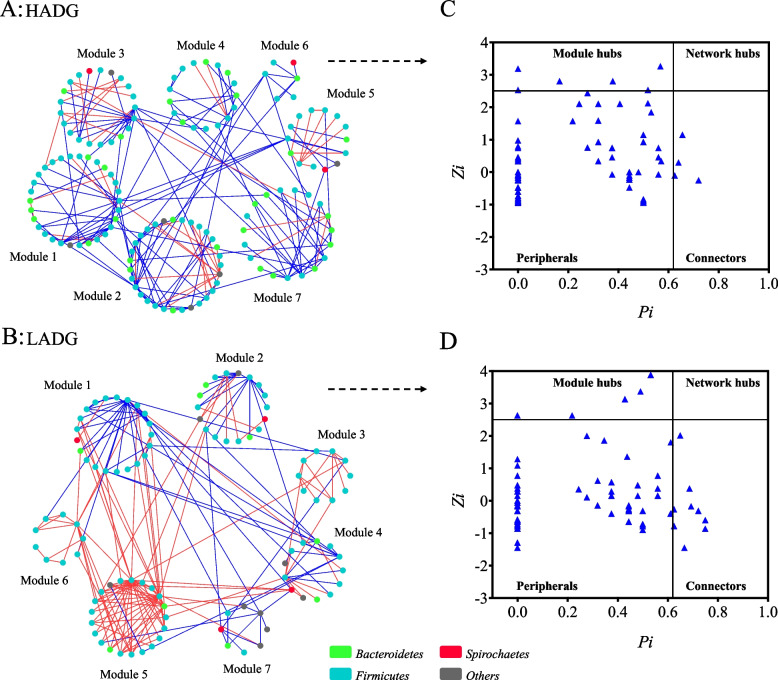
Co-occurrence network of ASVs, and distribution of ASVs based on their network roles in HADG and LADG goats (6-month-old, *n* = 10 each group). Nodes represent ASVs, and the color of connection lines between two nodes represents a positive (red) or a negative (blue) correlation (Pearson’s correlation, *P* < 0.05). No network hubs were identified in networks from both groups

The differentiated keystone microbial populations between HADG and LADG goats (6-month-old, *n* = 10 each group) were identified using the RMT-based network analysis. As shown in Fig. 2 and Additional file 1: Table S3, more than 85% of the ASV nodes in both networks were peripherals, which had only a few links and almost always to the nodes within their modules. A total of 10 ASVs of HADG goats, including two nodes (ASV5025 and ASV5719), assigned to HADG-related genera unclassified Prevotellaceae, and *Faecalibacterium* respectively, acted as connector species, linking modules together. Five ASVs were module hubs, such as ASV4418, assigned to HADG-related genera *Succinivibrio*, and may play an important role for the coherence of its own module. In the LADG network, the ASV that acted as connectors and module hubs were completely different compared to those in the HADG network. There were 9 ASV connectors in the LADG network, and three of them (ASV3231, ASV2741 and ASV2908) belonged to *Christensenellaceae R-7* group (LADG-enriched genus). Moreover, the relationship between ADG-related bacteria was analyzed (Additional file 1: Table S4 and Additional file 2: Fig. S1). At the genus level, those ADG-related bacteria had strong interactions with each other. *Prevotellaceae NK3B31* group, unclassified Prevotellaceae, *Christensenellaceae R-7* group, *Colidextribacter* had more than 12 links with other bacteria (*P* < 0.05).

### ADG-related bacterial abundance is correlated with rectum SCFA concentration

**Fig. 3:**
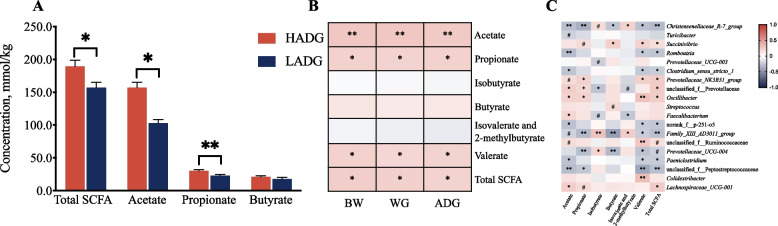
Rectum SCFA and their relationships with ADG-related microbiota. **A** Concentrations of SCFA in HADG and LADG goats (6-month-old, *n* = 10 each group). Significant differences were tested by *t*-test. The bars represent SEM. **B** Correlations between the rectum SCFA and growth rate of all young goats (*n* = 76, Spearman’s correlation). **C** Spearman’s rank correlations between ADG-related microbiota (relative abundance > 0.1%) and rectum SCFA (*n* = 76). The color gradient (in **B** and **C**) represents the values of correlation coefficients. ^#^*P* < 0.1, ^*^P < 0.05, ^**^P < 0.01

Compared to LADG goats, HADG goats had higher concentrations of total SCFA, acetate, propionate, and valerate in feces (*P* < 0.05, Fig. 3A, Additional file 2: Fig. S2A). The fecal concentrations of acetate, propionate, valerate, and total SCFA were positively correlated with the growth rate of young goats (*P* < 0.05, Fig. 3B). While, the ammonia nitrogen and lactate concentrations were not correlated with the growth rate of goats (Additional file 1: Table S5 and Additional file 2: Fig. S2) (*P* > 0.05).

The HADG-enriched bacteria, unclassified Prevotellaceae, and *Oscillibacter* were positively correlated with the total SCFA, acetate, and propionate concentrations (*P* < 0.05, Fig. 3C). *Prevotellaceae NK3B31* group was positively correlated with the propionate, and total SCFA concentrations (*P* < 0.05). *Lachnospiraceae UCG-001* was positively correlated with the total SCFA concentration. In contrast, some LADG-enriched bacteria were significantly negatively correlated with the total SCFA, and two major microbial components, such as *Christensenellaceae R-7* group, unclassified Peptostreptococcaceae, were negatively correlated with the concentrations of total SCFA, acetate, and propionate. *Clostridium* sensu stricto 1 and *Romboutsia* were negatively correlated with the total SCFA and acetate concentrations (*P* < 0.05).

### The correlations between microbial network modules and rectum SCFA

**Fig. 4:**
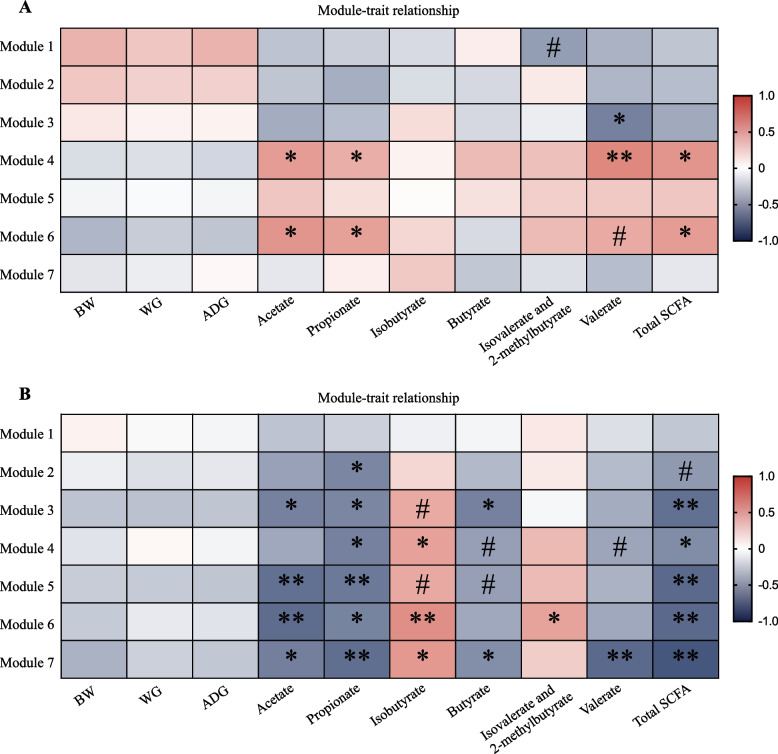
Network analysis on the rectum microbiota modules and growth rate, and rectum SCFA in HADG (**A**) and LADG (**B**) goats (6-month-old, *n* = 10 each group). The color gradient represents the values of correlation coefficients (spearman’s correlation). ^#^*P* < 0.1, ^*^P < 0.05, ^**^P < 0.01

The network module-trait relationships were investigated using a Pearson correlation analysis to understand the relationship between individual modules and rectum SCFA (Fig. 4). In the LADG network, at least five modules were correlated with the total SCFA, and/or acetate, and propionate (Fig. 4B, *P* < 0.05). Module #3, 5, 6, and 7 were negatively correlated with the total SCFA, acetate and propionate concentrations (*P* < 0.05). Module #4 was negatively correlated with the propionate and total SCFA concentrations (*P* < 0.05). A notable attribution of two modules (#3, and 5) was that the majority of nodes belong to the LADG-enriched genera, including *Christensenellaceae R-7* group, *Turicibacter*, and *Romboutsia*. In contrast, in the HADG network, module #6 was positively correlated with the concentrations of acetate, propionate, and total SCFA (*P* < 0.06, Fig. 4A).

Together, microbiota and microbial interactions in the rectum had effects on feed degradation and SCFA synthesis, and subsequently affected the energy supply and host growth performances in young goats.

### The rectum microbiota and their metabolites are linked to animal immunity

**Fig. 5:**
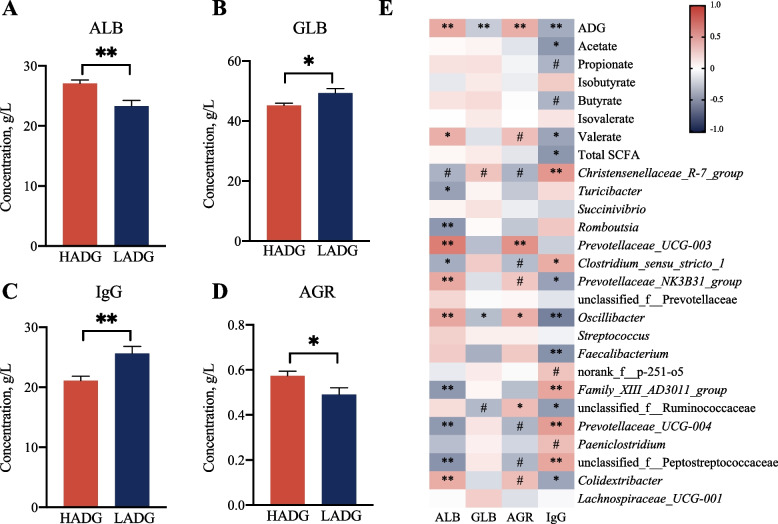
Difference in serum immune parameters between HADG and LADG goats (6-month-old, *n* = 10 each group), and the relationship between these immune parameters and ADG-related rectum microbiota. **A** albumin (ALB), (**B**) globulin (GLB), (**C**) IgG, (**D**) the ALB to GLB ratio (ARG), significant differences were tested by *t-*test. The bars represent SEM. **E** Correlations between serum immune parameters and ADG-related microbiota, rectum SCFA of HADG and LADG (Spearman’s correlation). The color gradient represents the values of correlation coefficients. ^#^*P* < 0.1, ^*^P < 0.05, ^**^P < 0.01

As shown above, most of those ADG-related bacteria were significantly correlated with SCFA biosynthesis, which are critical for both gut immunity and animal growth [31]. Then we analyzed the differences in the serum ALB, GLB, and IgG concentration and AGR between HADG and LADG young goats (*n* = 10 each group, Fig. 5). The results showed that HADG goats had the greater ALB concentration and AGR value, but the lower GLB and IgG concentrations (*P* < 0.05). Furthermore, Spearman correlation analysis showed that serum IgG was significantly negatively correlated with ADG and acetate and total SCFA concentrations, and negatively with the abundances of those HADG-related bacteria, such as *Prevotellaceae NK3B31* group, *Oscillibacter*, *Faecalibacterium*, and *Colidextribacter* (*P* < 0.05). While serum IgG was significantly positively correlated with the abundances of LADG-related bacteria, such as *Christensenellaceae R-7* group and *Clostridium* sensu stricto 1. Meanwhile, the serum IgG had weak negative correlations with the concentrations of propionate and butyrate (*P* < 0.1).

### Rectum microbiota and SCFA with high prediction accuracy for classifying host growth rate

**Fig. 6:**
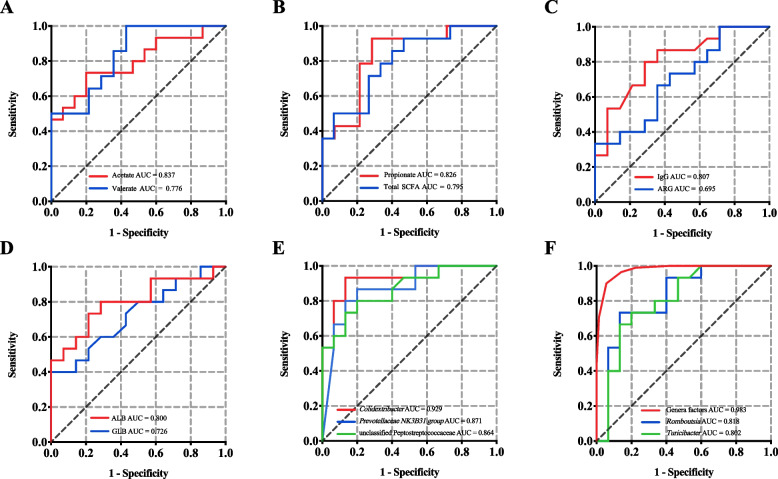
Classification analyses based on random forest model. Classification of host ADG (HADG vs. LADG) using rectum SCFA (**A**, **B**), serum immune factors (**C**, **D**), and rectum microbiota (**E**, **F**)

We used the rectum microbiota and SCFA, and serum immune parameters to classify young goats with different growth rates by using the random forest model (Fig. 6). The concentrations of acetate, propionate, and total SCFA could classify the HADG and LADG goats with high accuracy (AUC > 0.77, Fig. 6A, B). Among these 4 immune parameters, serum IgG and ALB concentrations had the AUC values > 0.80 in classifying HADG and LADG goats (Fig. 6C, D). Among the rectum bacteria (Fig. 6E, F), *Colidextribacter*, *Prevotellaceae NK3B31* group, unclassified Peptostreptococcaceae, *Romboutsia*, *Turicibacter* were the top 5 genera with the AUC values > 0.80 in classifying HADG and LADG goats. Furthermore, six genera, including *Colidextribacter*, *Prevotella*, *Prevotellaceae NK3B31* group, *Lachnospiraceae UCG-001*, *Oscillibacter*, *Prevotellaceae UCG-003*, were selected by the random forest model with MDA > 2.0, and the ROC curve of those bacteria combination represented an AUC of 0.983.

### Differences in rectum microbiota composition and SCFA between young and adult goats

A few of differences in rectum microbiota diversity, structure, composition, and SCFA synthesis between HAL and LAL goats (19-month-old, *n* = 8 each group) were detected (Additional file 2: Fig. S3–S6).

**Fig. 7:**
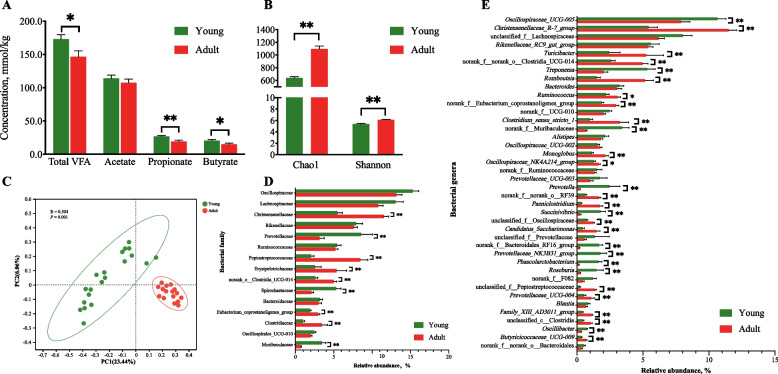
Differential rectum microbiota diversity, structure and composition between young (6-month-old, *n* = 20) and adult (19-month-old, *n* = 16) goats. **A** Concentrations of SCFA between young and adult goats. Significant differences were tested by *t*-test. **B** The Chao1 and Shannon indexes of rectum microbiota between young and adult goats. **C** Principal Coordinate Analysis (PCoA) of recutm microbiota at the ASV level based on the Bray-Curtis dissimilarity. The 15 most abundance bacterial family (**D**) and 50 most abundant bacterial genera (**E**). The differences in data in (**B**), (**D**), and (**E**) were assessed by the Wilcon rank-sum test. The bars represent SEM. ^*^P < 0.05, ^**^P < 0.01

Then the differences in microbiota features were analyzed between young (6-month-old) and adult (19-month-old) goats (Fig. 7). Compared to those in young goats, the concentrations of rectum total SCFA, propionate, and butyrate were significantly decreased (Fig. 7A), the rectum microbiota richness (Chao 1 index) and diversity (Shannon index) were significantly greater in adult goats (Fig. 7B). The communities of rectum microbiota in adult goats were more convergent and clearly distinguished from those in young goats (ANOSIM *r* = 0.504, *P* = 0.001, Fig. 7C).

At the family level, Christensenellaceae, Peptostreptococcaceae, Erysipelotrichaceae, norank Clostridia UCG-014, *Eubacterium coprostanoligenes* group, Clostridiaceae were more abundant in adult goats than those in young goats, while Prevotellaceae, Spirochaetaceae, Muribaculaceae showed a opposite trend (*P* < 0.05). At the genus level, 32 of the 50 most abundant bacterial genera were significantly different between the adult and young goats (*P* < 0.05); of these, 20 genera, including the abundances of *Christensenellaceae R-7* group, *Turicibacter*, *Romboutsia*, and *Clostridium* sensu stricto 1, were significantly increased in the adult goats, compared to those in the young goats. Twelve genera, including unclassified *Prevotellaceae*, *Succinivibrio*, *Prevotellaceae NK3B31* group, and *Oscillibacter* were significantly decreased in the adult goats. The results showed that those HADG-enriched bacteria decreased and the LADG-enriched bacteria increased with maturity of goats.

PICRUSt2 was employed to predict the function of the rectum microbiota (Additional file 1: Table S6). Comparison with the young goats, the adult goats had a significantly lower relative abundance for Metabolism and Organismal Systems-related genes, but a higher relative abundance of genes relating to Genetic Information Processing, Environmental Information Processing, and Cellular Processes in multiple KEGG (level 1) categories. Compared with the young goats, the adult goats had a lower relative abundance of genes relating to the metabolism of carbohydrate, glycan, and amino acids, and Cell growth and death, Environmental adaptation, Transport and Catabolism. Whereas it had a higher relative abundance of genes relating to Replication and repair, Signal transduction, Cellular community (prokaryotes), Cell motility, Nervous system, and Transcription.

Together, with the maturation of goats, the function of rectum microbiota became more diversified. However, the contribution of the changes of those microbiota to feed degradation and energy supply decreased in adult goats.

## Discussion

Recent studies showed that rumen microbiome was closely associated with productivity and health of host [32, 33]. More and more studies are addressing the compositions and functions of gut microbiome in ruminant animals [34, 35]. However, the relationships between hindgut microbiota and the growth performance of young ruminants remain unclear. In the present study, a total of 76 young goats, which were fed the same diet and raised under the same condition, were investigated. Based on the comparisons between the different growth rates or growth stage goats and the analysis of the correlation of large samples and microbial co-occurrence network, we found that some keystone rectum microbiota, played more critical roles in early life than in adulthood, especially in modulating the rectum fermentation and animal growth.

The significant differences in the microbial community composition of fecal samples were found between HADG and LADG goats in the present study. Moreover, the analysis of microbial co-occurrence network showed that the HADG-related bacteria (unclassified Prevotellaceae [0.86%, 29/306], *Succinivibrio* [2.65%, 10/306], and *Faecalibacterium* [0.43%, 41/306]), and LADG-related bacteria [*Christensenellaceae R-7* group (5.88%, 4/306)], which were the top 50 genera of the total 306 genera measured of all young goats, had significantly interacted with other rectum bacteria. These taxa may play momentous roles as functional-keystone bacteria in shaping the hindgut microbial community and improving feed digestion. In the present study, we mainly focused on the relationship between “common” bacteria (relative abundance > 0.1%), gut fermentation and animal performance. Future studies should explore the function of gut “rare” species in ecosystem multifunctionality.

In the present study, we found the concentrations of acetate, propionate, and total SCFA in feces were significantly and positively correlated with ADG of young goats. Carbohydrates are the important sources of energy for ruminants and microbial cells. Gut microbes are involved in the digestion, absorption, and metabolism of the plant polysaccharides in the gastrointestinal tract, and the end products, SCFA, mainly consist of acetate, propionate, and butyrate, which have profound effects on host productivity [36]. Those HADG-enriched bacteria identified in this study are generally considered as SCFA producing and beneficial bacteria. Prevotellaceae family and its three genera (*Prevotellaceae UCG-003*, *Prevotellaceae NK3B31* group, and unclassified Prevotellaceae) can utilize fiber as substrates for produce primarily acetate, succinate, and propionate [37], showing a positive correlation with the goat growth rate. *Succinivibrio* is a predominant and important propionate producing genus, which is associated with the feed efficiency [38–40]. *Oscillibacter*, *Faecalibacterium,* and *Colidextribacter* can produce SCFA (mainly butyrate), contributing primarily to complex sugar degradation [41–44]. It has been reported that those 3 genera improved the gut health and were decreased in different intestinal disorders, such as Crohn’s disease and diarrhea [45–47]. *Streptococcus* is involved in amino acid biosynthesis and the metabolism of energy substrates [48–50], and a recent study showed that *Streptococcus* may promote pig growth performance [12]. These previous studies support our findings that those HADG-related bacteria were positively correlated with rectum SCFA production.

The family Christensenellaceae had a higher abundance of LADG goats than that in HADG goats in the present study, which is supported by a report that this taxon was enriched in individuals with low body mass index [51]. Human individuals with a high abundance of Christensenellaceae had low lipid biosynthesis and energy metabolism pathway, which may explain the negative association between the body weight and Christensenellaceae abundance [52, 53]. Meanwhile, the proportions of *Turicibacter* and *Romboutsia*, which were positively correlated to colitis in both mice and humans [54, 55], increased in LADG goats in this study. Future research activities will be carried out to comprehensively understand how those growth-related/keystone bacteria influence microbial community, and effective microbial manipulation means and techniques can be developed to improve feed efficiency and animal performance.

In the present study, several HADG-related bacteria, such as *Prevotellaceae NK3B31* group, *Oscillibacter*, and *Faecalibacterium*, showed positive correlations with fecal total SCFA concentration and negatively associated with serum IgG level; and the acetate, propionate, butyrate, and total SCFA concentrations were negatively correlated with the IgG level. A study showed that manipulating the gut microbial composition in early life with oral supplementation of *Faecalibacterium* spp. could decrease calf diarrhea [47] and improve calf health and growth [56]. Taking the finding of the present study and previous studies together, enrich of those SCFA-producing bacteria in the gut likely inhibit growth of pathogenic bacteria, suppress inflammation, and promote animal growth, where SCFA can decrease intestinal permeability and circulating endotoxins, lower inflammation and oxidative stress, and play important roles in anti-inflammatory function [31, 57]. Therefore, research in the future should focus on developing probiotics that promote the production of more SCFA and inhibit the pathogenic bacterial taxa in the gut.

The random forest classification is an effective tool to learn approaches of the microbiota and their metabolites for prediction of host phenotypes, including for the prediction of disease risk [58–60] and productive performance [38, 61]. In the present study, we found that the acetate concentrate in feces could classify HADG and LADG goats with an accuracy of 83.7%. Notably, six genera (*Colidextribacter*, *Prevotella*, *Prevotellaceae NK3B31* group, *Lachnospiraceae UCG-001*, *Oscillibacter*, and *Prevotellaceae UCG-003*) all together could predict the host growth rate with an accuracy of 98.3%. Further studies are warranted to test the robustness of these potential markers, which can help us to apply rectum bacterial community and their metabolites for predicting the growth performance of goats in practice.

Studies on gut [62, 63] and rumen [64] microbiomes of ruminant animals have revealed that individual variations are greater in early life than those in adulthood, suggesting the establishment of a more similar microbial community in adult animals. This is consistent with the finding in this experiment that there were few differences in rectum microbiota features (diversity, structure, composition) and fermentation between adult HADG and LADG goats. While, compared to the young goats, the HADG-related bacteria, such as family Prevotellaceae, genera unclassified Prevotellaceae, *Succinivibrio*, *Prevotellaceae NK3B31* group, and *Oscillibacter*, which are SCFA-producing bacteria, were decreased in the adult goats. Moreover, adult goats had lower fecal SCFA concentrations and microbial genes related to carbohydrate and protein metabolisms. Fan et al. discovered that more bacteria in pre-weaning calves were associated with the weight gain of calves than the correlation in the fattening stages [65]. Taken together, individual variation in the rectum microbial community became low in adult goats, and the contribution of the rectum microbiota to feed efficiency and energy supply declined. These findings are consistent with the results that hindgut microbiota play more critical role in hindgut fermentation when the rumen is not fully developed [16]. Therefore, microbial interventions in the gut of early may be one option to obtain a desirable and healthy gut microbiome, which helps to improve the gut fermentation and animal performance in future.

Host genetics play an important role in the establishment and shaping of the gut microbiota, as it has been demonstrated that the composition of bacterial community is influenced by specific host genomic loci [51, 66]. Furthermore, a recent study reported that individual variations in gut microbiota of calves were primarily explained by genetic differences among the hosts [67]. All goats used in this study were fed the same diet, kept under the same condition, and housed together from birth to minimize nongenetic influences to the gut microbiota. Therefore, those ADG-related microbiota taxa in young goats must have been attributed to genetic variations of animals. In other words, the gut microbial community composition could be inheritable to a certain extent. For example, the family Christensenellaceae was reported to be the most heritable microbial taxon in a human study [51]. Future studies will explore how host genotypes modulate gut bacterial composition, which could further be applied to manipulate the gut microbiome through selective breeding of the hosts and to improve animal feed efficiency and performance.

## Conclusion

In conclusion, the rectum microbial variations can affect the rectum fermentation and change the health and growth performance of young goats. Some specific rectum bacteria, such as unclassified Prevotellaceae, *Succinivibrio*, and *Faecalibacterium*, play keystone roles in shaping rectum microbial community, and these SCFA- producing taxa support more SCFA synthesis for a high growth rate of goats. Moreover, rectum microbiota of young goats could become effective biomarkers for predicting animal growth performance. The present study also showed that the individual variation in rectum microbiota was relative higher in early life of goats and played a more important role in rectum fermentation than that in adult animals. These findings can help better understanding how the hindgut microbiota affect the health and growth performance of young ruminants, and provide a foundation for further studies on early microbial interventions for improving animal health and performance.

## Supplementary Information

**Additional file 1: Table S1.** The feeding programs of the experiment goats. **Table S2.** The relationship between rectum microbiota (relative abundance > 0.1%) and ADG of young goats (*n* = 76). **Table S3.** The topological roles of ASV in the HADG and LADG network. **Table S4.** The relationship between ADG-related bacteria in all young goats (*n* = 76, Spearman’s correlation, *P* < 0.05). **Table S5.** The relationship between NH_3_-N, lactate and growth performance traits of young goats (*n* = 76). **Table S6.** Prediction of the differential function of rectum microbiota between young goats (*n* = 20) and adult goats (*n* = 16) in KEGG pathways based on PICRUSt2. The *P* value was calculated based on the Wilcoxon rank-sum test.

**Additional file 2: Fig. S1.** The relationship between ADG-related bacterial genera. Connections were detected based on Spearman’s rank correlations (*P* < 0.05). Dot size represent number of connections with other taxa. Dot color represents the relationship between the relative abundance of bacterial genera and ADG. Edge color represents either positive or negative associations between bacteria. **Fig. S2.** The difference in rectum minor SCFA, NH_3_-N and lactate between HADG and LADG group (6-month-old, *n* = 10 each group). Differences in data were assessed by student t test, The bars represent mean ± SEM. ** *P* < 0.01. **Fig. S3.** The difference in rectum microbiota diversity and structure between HAL and LAL group (16-month-old, *n* = 8 each group). Significant differences were tested by Wilcoxon rank-sum test. The bars represent mean ± SEM. HAL: adult HADG goats, LAL: adult LADG goats. **Fig. S4.** The difference in rectum SCFA between HAL and LAL group (16-month-old, *n* = 8 each group). Significant differences were tested by *t* test. The bars represent mean ± SEM. HAL: adult HADG goats, LAL: adult LADG goats. **Fig. S5.** The difference in 15 most abundant rectum bacterial family between HAL and LAL group (19-month-old, *n* = 8 each group). Significant differences were tested by Wilcoxon rank-sum test. The bars represent mean ± SEM. ^*^P < 0.05. HAL: adult HADG goats, LAL: adult LADG goats. **Fig. S6.** The difference in 50 most abundant hindgut bacterial genera between adult HADG and LADG group (19-month-old, *n* = 8 each group). Significant differences were tested by Wilcoxon rank-sum test. The bars represent mean ± SEM. HAL: adult HADG goats, LAL: adult LADG goats.

## Data Availability

Illumina data are available at NCBI (BioProject ID: PRJNA871392).

## Abbreviations

ADG: Daily weight gain
AGR: Albumin to globulin ratio
ALB: Albumin
ANOSIM: Analysis of similarities
ASV: Amplicon sequence variant
AUC: Area under curve
DMI: Dry matter intake
GLB: Globulin
KEGG: Kyoto encyclopedia of genes and genomes
MDA: Mean decrease accuracy
PCoA: Principal coordinate analysis
PICRUSt: Phylogenetic investigation of communities by reconstruction of unobserved states
Pi: Among-module connectivity
RMT: Random matrix theory
ROC: Receiver operating characteristic
SCFA: Short-chain fatty acids
Zi: Within-module degree

## References

[1] Shabat SKB, Sasson G, Doron-Faigenboim A, Durman T, Yaacoby S, Berg Miller ME (2016). Specific microbiome-dependent mechanisms underlie the energy harvest efficiency of ruminants. ISME J.

[2] Wallace RJ, Sasson G, Garnsworthy PC, Tapio I, Gregson E, Bani P, et al. A heritable subset of the core rumen microbiome dictates dairy cow productivity and emissions. Sci Adv. 2019;5(7):eaav8391. 10.1126/sciadv.aav8391. 10.1126/sciadv.aav8391 PMC6609165 31281883

[3] Xue MY, Sun HZ, Wu XH, Liu JX. Multi-omics reveals that the rumen microbiome and its metabolome together with the host metabolome contribute to individualized dairy cow performance. Microbiome. 2020;8:64. 10.1186/s40168-020-00819-8. 10.1186/s40168-020-00819-8 PMC7218573 32398126

[4] Meale SJ, Li S, Azevedo P, Derakhshani H, DeVries T, Plaizier J (2017). Weaning age influences the severity of gastrointestinal microbiome shifts in dairy calves. Sci Rep.

[5] Malmuthuge N (2017). Understanding the gut microbiome of dairy calves: opportunities to improve early-life gut health. J Dairy Sci.

[6] Clemente JC, Ursell LK, Parfrey LW, Knight R (2012). The impact of the gut microbiota on human health: an integrative view. Cell.

[7] Fan Y, Pedersen O (2021). Gut microbiota in human metabolic health and disease. Nat Rev Microbiol.

[8] Gentile CL, Weir TL (2018). The gut microbiota at the intersection of diet and human health. Science.

[9] Kogut MH, Arsenault RJ (2016). Gut health: the new paradigm in food animal production. Front Vet Sci.

[10] Hu J, Ma L, Nie Y, Chen J, Zheng W, Wang X (2018). A microbiota-derived bacteriocin targets the host to confer diarrhea resistance in early-weaned piglets. Cell Host Microbe.

[11] Chen C, Fang S, Wei H, He M, Fu H, Xiong X, et al. *Prevotella copri* increases fat accumulation in pigs fed with formula diets. Microbiome. 2021;9:175. 10.1186/s40168-021-01110-0. 10.1186/s40168-021-01110-0 PMC8380364 34419147

[12] Wang X, Tsai T, Deng F, Wei X, Chai J, Knapp J, et al. Longitudinal investigation of the swine gut microbiome from birth to market reveals stage and growth performance associated bacteria. Microbiome. 2019;7:109. 10.1186/s40168-019-0721-7. 10.1186/s40168-019-0721-7 PMC6664762 31362781

[13] Malmuthuge N, Guan LL (2016). Gut microbiome and omics: a new definition to ruminant production and health. Anim Front.

[14] Oikonomou G, Teixeira AGV, Foditsch C, Bicalho ML, Machado VS, Bicalho RC (2013). Fecal microbial diversity in pre-weaned dairy calves as described by pyrosequencing of metagenomic 16S rDNA. Associations of *Faecalibacterium* species with health and growth. PLoS One.

[15] Kim HS, Whon TW, Sung H, Jeong YS, Jung ES, Shin NR, et al. Longitudinal evaluation of fecal microbiota transplantation for ameliorating calf diarrhea and improving growth performance. Nat Commun. 2021;12:161. 10.1038/s41467-020-20389-5. 10.1038/s41467-020-20389-5 PMC7794225 33420064

[16] Gressley T, Hall M, Armentano L (2011). Ruminant nutrition symposium: productivity, digestion, and health responses to hindgut acidosis in ruminants. J Anim Sci.

[17] Canfora EE, Jocken JW, Blaak EE (2015). Short-chain fatty acids in control of body weight and insulin sensitivity. Nat Rev Endocrinol.

[18] Yang W, Yu T, Huang X, Bilotta AJ, Xu L, Lu Y, et al. Intestinal microbiota-derived short-chain fatty acids regulation of immune cell IL-22 production and gut immunity. Nat Commun. 2020;11:4457. 10.1038/s41467-020-18262-6. 10.1038/s41467-020-18262-6 PMC7478978 32901017

[19] McKay Z, Lynch M, Mulligan F, Rajauria G, Miller C, Pierce K (2019). The effect of concentrate supplementation type on milk production, dry matter intake, rumen fermentation, and nitrogen excretion in late-lactation, spring-calving grazing dairy cows. J Dairy Sci.

[20] Magoč T, Salzberg SL (2011). FLASH: fast length adjustment of short reads to improve genome assemblies. Bioinformatics.

[21] Chen S, Zhou Y, Chen Y, Gu J (2018). Fastp: an ultra-fast all-in-one FASTQ preprocessor. Bioinformatics.

[22] Callahan BJ, McMurdie PJ, Rosen MJ, Han AW, Johnson AJA, Holmes SP (2016). DADA2: high-resolution sample inference from Illumina amplicon data. Nat Methods.

[23] Douglas GM, Maffei VJ, Zaneveld JR, Yurgel SN, Brown JR, Taylor CM (2020). PICRUSt2 for prediction of metagenome functions. Nat Biotechnol.

[24] Li F, Yang X, Cao Y, Li S, Yao J, Li Z (2014). Effects of dietary effective fiber to rumen degradable starch ratios on the risk of sub-acute ruminal acidosis and rumen content fatty acids composition in dairy goat. Anim Feed Sci Technol.

[25] Broderick G, Kang J (1980). Automated simultaneous determination of ammonia and total amino acids in ruminal fluid and *in vitro* media. J Dairy Sci.

[26] Fan Y, Li X, Xu Q, Zhang Y, Yang X, Han X (2020). Serum albumin mediates the effect of multiple per-and polyfluoroalkyl substances on serum lipid levels. Environ Pollut.

[27] Deng Y, Jiang YH, Yang Y, He Z, Luo F, Zhou J. Molecular ecological network analyses. BMC Bioinform. 2012;13:1. 10.1186/1471-2105-13-113. 10.1186/1471-2105-13-113 PMC3428680 22646978

[28] Zhou J, Deng Y, Luo F, He Z, Tu Q, Zhi X (2010). Funiction molecular ecological networks. mBio.

[29] Zhou J, Deng Y, Luo F, He Z, Yang Y (2011). Phylogenetic molecular ecological network of soil microbial communities in response to elevated CO_2_. mBio.

[30] Breiman L (2001). Random forests. Mach Learn.

[31] Koh A, De Vadder F, Kovatcheva-Datchary P, Bäckhed F (2016). From dietary fiber to host physiology: short-chain fatty acids as key bacterial metabolites. Cell.

[32] Huws SA, Creevey CJ, Oyama LB, Mizrahi I, Denman SE, Popova M (2018). Addressing global ruminant agricultural challenges through understanding the rumen microbiome: past, present, and future. Front Microbiol.

[33] Mizrahi I, Wallace RJ, Moraïs S (2021). The rumen microbiome: balancing food security and environmental impacts. Nat Rev Microbiol.

[34] O’Hara E, Neves AL, Song Y, Guan LL (2020). The role of the gut microbiome in cattle production and health: driver or passenger?. Annu Rev Anim Biosci.

[35] Arshad MA, Hassan F-u, Rehman MS, Huws SA, Cheng Y, Din AU (2021). Gut microbiome colonization and development in neonatal ruminants: strategies, prospects, and opportunities. Anim Nutr.

[36] Tremaroli V, Bäckhed F (2012). Functional interactions between the gut microbiota and host metabolism. Nature.

[37] Shah HN, Chattaway MA, Rajakurana L, Gharbia SE, Trujillo ME, Dedysh S, DeVos P, Hedlund B, Kämpfer P, Rainey FA (2015). Prevotella. Bergey's manual of systematics of archaea and bacteria.

[38] Xue MY, Xie YY, Zhong Y, Ma XJ, Sun HZ, Liu JX. Integrated meta-omics reveals new ruminal microbial features associated with feed efficiency in dairy cattle. Microbiome. 2022;10:32. 10.1186/s40168-022-01228-9. 10.1186/s40168-022-01228-9 PMC8849036 35172905

[39] Zhang Y, Zhang X, Li F, Li C, Li G, Zhang D (2021). Characterization of the rumen microbiota and its relationship with residual feed intake in sheep. Animal..

[40] Hernandez-Sanabria E, Goonewardene LA, Wang Z, Durunna ON, Moore SS, Guan LL (2012). Impact of feed efficiency and diet on adaptive variations in the bacterial community in the rumen fluid of cattle. Appl Environ Microbiol.

[41] Lee GH, Kumar S, Lee JH, Chang DH, Kim DS, Choi SH (2012). Genome sequence of *Oscillibacter ruminantium* strain GH1, isolated from rumen of Korean native cattle. J Bacteriol.

[42] Iino T, Mori K, Tanaka K, Suzuki K-i, Harayama S (2007). *Oscillibacter valericigenes* gen. Nov., sp. nov., a valerate-producing anaerobic bacterium isolated from the alimentary canal of a Japanese corbicula clam. Int J Syst Evol Microbiol.

[43] Barcenilla A, Pryde SE, Martin JC, Duncan SH, Stewart CS, Henderson C (2000). Phylogenetic relationships of butyrate-producing bacteria from the human gut. Appl Environ Microbiol.

[44] Wang Q, Wang C, Abdullah, Tian W, Qiu Z, Song M, et al. Hydroxytyrosol alleviates dextran sulfate sodium-induced colitis by modulating inflammatory responses, intestinal barrier, and microbiome. J Agric Food Chem. 2022;70(7):2241–52. 10.1021/acs.jafc.1c07568. 10.1021/acs.jafc.1c07568 35133830

[45] Rettedal EA, Gumpert H, Sommer MO. Cultivation-based multiplex phenotyping of human gut microbiota allows targeted recovery of previously uncultured bacteria. Nat Commun. 2014;5:4714. 10.1038/ncomms5714. 10.1038/ncomms5714 25163406

[46] Liang J, Kou S, Chen C, Raza SHA, Wang S, Ma X, et al. Effects of *Clostridium butyricum* on growth performance, metabonomics and intestinal microbial differences of weaned piglets. BMC Microbiol. 2021;21:85. 10.1186/s12866-021-02143-z. 10.1186/s12866-021-02143-z PMC7983215 33752593

[47] Lopez-Siles M, Duncan SH, Garcia-Gil LJ, Martinez-Medina M (2017). *Faecalibacterium prausnitzii*: from microbiology to diagnostics and prognostics. ISME J.

[48] Ogunade I, Schweickart H, McCoun M, Cannon K, McManus C (2019). Integrating 16S rRNA sequencing and LC–MS-based metabolomics to evaluate the effects of live yeast on rumen function in beef cattle. Animals.

[49] Jin D, Zhao S, Wang P, Zheng N, Bu D, Beckers Y (2016). Insights into abundant rumen ureolytic bacterial community using rumen simulation system. Front Microbiol.

[50] Kakimoto S, Okazaki K, Sakane T, Imai K, Sumino Y, Akiyama S-i (1989). Isolation and taxonomie characterization of acid urease-producing bacteria. Agric Biol Chem.

[51] Goodrich JK, Waters JL, Poole AC, Sutter JL, Koren O, Blekhman R (2014). Human genetics shape the gut microbiome. Cell.

[52] Li X, Li Z, He Y, Li P, Zhou H, Zeng N (2020). Regional distribution of Christensenellaceae and its associations with metabolic syndrome based on a population-level analysis. PeerJ.

[53] Waters JL, Ley RE. The human gut bacteria Christensenellaceae are widespread, heritable, and associated with health. BMC Biol. 2019;17:83. 10.1186/s12915-019-0699-4. 10.1186/s12915-019-0699-4 PMC6819567 31660948

[54] Munyaka PM, Rabbi MF, Khafipour E, Ghia JE (2016). Acute dextran sulfate sodium (DSS)-induced colitis promotes gut microbial dysbiosis in mice. J Basic Microbiol.

[55] Wang HG, Zhang MN, Wen X, He L, Zhang MH, Zhang JL, et al. Cepharanthine ameliorates dextran sulphate sodium-induced colitis through modulating gut microbiota. Microb Biotechnol. 2022. 10.1111/1751-7915.14059. 10.1111/1751-7915.14059 PMC9328732 35439340

[56] Foditsch C, Pereira RVV, Ganda EK, Gomez MS, Marques EC, Santin T, et al. Oral administration of *Faecalibacterium prausnitzii* decreased the incidence of severe diarrhea and related mortality rate and increased weight gain in preweaned dairy heifers. PLoS One. 2015;10(12):e0145485. 10.1371/journal.pone.0145485. 10.1371/journal.pone.0145485 PMC4692552 26710101

[57] Littman DR, Pamer EG (2011). Role of the commensal microbiota in normal and pathogenic host immune responses. Cell Host Microbe.

[58] Islam J, Tanimizu M, Shimizu Y, Goto Y, Ohtani N, Sugiyama K, et al. Development of a rational framework for the therapeutic efficacy of fecal microbiota transplantation for calf diarrhea treatment. Microbiome. 2022;10:31. 10.1186/s40168-021-01217-4. 10.1186/s40168-021-01217-4 PMC8858662 35184756

[59] Touw WG, Bayjanov JR, Overmars L, Backus L, Boekhorst J, Wels M (2013). Data mining in the life sciences with random forest: a walk in the park or lost in the jungle?. Brief Bioinform.

[60] Cao Y, Wang L, Ke S, Gálvez JAV, Pollock NR, Barrett C (2021). Fecal mycobiota combined with host immune factors distinguish *Clostridioides difficile* infection from asymptomatic carriage. Gastroenterology.

[61] Deehan EC, Zhang Z, Riva A, Armet AM, Perez-Muñoz ME, Nguyen NK, et al. Elucidating the role of the gut microbiota in the physiological effects of dietary fiber. Microbiome. 2022;10:77. 10.1186/s40168-022-01248-5. 10.1186/s40168-022-01248-5 PMC9107176 35562794

[62] Klein-Jöbstl D, Schornsteiner E, Mann E, Wagner M, Drillich M, Schmitz-Esser S (2014). Pyrosequencing reveals diverse fecal microbiota in Simmental calves during early development. Front Microbiol.

[63] Yatsunenko T, Rey FE, Manary MJ, Trehan I, Dominguez-Bello MG, Contreras M (2012). Human gut microbiome viewed across age and geography. Nature.

[64] Jami E, Israel A, Kotser A, Mizrahi I (2013). Exploring the bovine rumen bacterial community from birth to adulthood. ISME J.

[65] Fan P, Nelson CD, Driver JD, Elzo MA, Peñagaricano F, Jeong KC (2021). Host genetics exerts lifelong effects upon hindgut microbiota and its association with bovine growth and immunity. ISME J.

[66] Bonder MJ, Kurilshikov A, Tigchelaar EF, Mujagic Z, Imhann F, Vila AV (2016). The effect of host genetics on the gut microbiome. Nat Genet.

[67] Fan P, Bian B, Teng L, Nelson CD, Driver J, Elzo MA (2020). Host genetic effects upon the early gut microbiota in a bovine model with graduated spectrum of genetic variation. ISME J.

