# Supplementary material for: Potential roles of the rectum keystone microbiota in modulating the microbial community and growth performance in goat model
Source: J Anim Sci Biotechnol. 2023 Apr 7;14:55. doi: 10.1186/s40104-023-00850-3 (PMC10080759; doi:10.1186/s40104-023-00850-3)
Supplement: Supplementary file 2 — Additional file 2: Fig. S1. The relationship between ADG-related bacterial genera. Connections were detected based on Spearman’s rank correlations (P < 0.05). Dot size represent number of connections with other taxa. Dot color represents the relationship between the relative abundance of bacterial genera and ADG. Edge color represents either positive or negative associations between bacteria. Fig. S2. The difference in rectum minor SCFA, NH3-N and lactate between HADG and LADG group (6-month-old, n = 10 each group). Differences in data were assessed by student t test, The bars represent mean ± SEM. ** P < 0.01. Fig. S3. The difference in rectum microbiota diversity and structure between HAL and LAL group (16-month-old, n = 8 each group). Significant differences were tested by Wilcoxon rank-sum test. The bars represent mean ± SEM. HAL: adult HADG goats, LAL: adult LADG goats. Fig. S4. The difference in rectum SCFA between HAL and LAL group (16-month-old, n = 8 each group). Significant differences were tested by t test. The bars represent mean ± SEM. HAL: adult HADG goats, LAL: adult LADG goats. Fig. S5. The difference in 15 most abundant rectum bacterial family between HAL and LAL group (19-month-old, n = 8 each group). Significant differences were tested by Wilcoxon rank-sum test. The bars represent mean ± SEM. *P < 0.05. HAL: adult HADG goats, LAL: adult LADG goats. Fig. S6. The difference in 50 most abundant hindgut bacterial genera between adult HADG and LADG group (19-month-old, n = 8 each group). Significant differences were tested by Wilcoxon rank-sum test. The bars represent mean ± SEM. HAL: adult HADG goats, LAL: adult LADG goats. [file 40104_2023_850_MOESM2_ESM.docx]

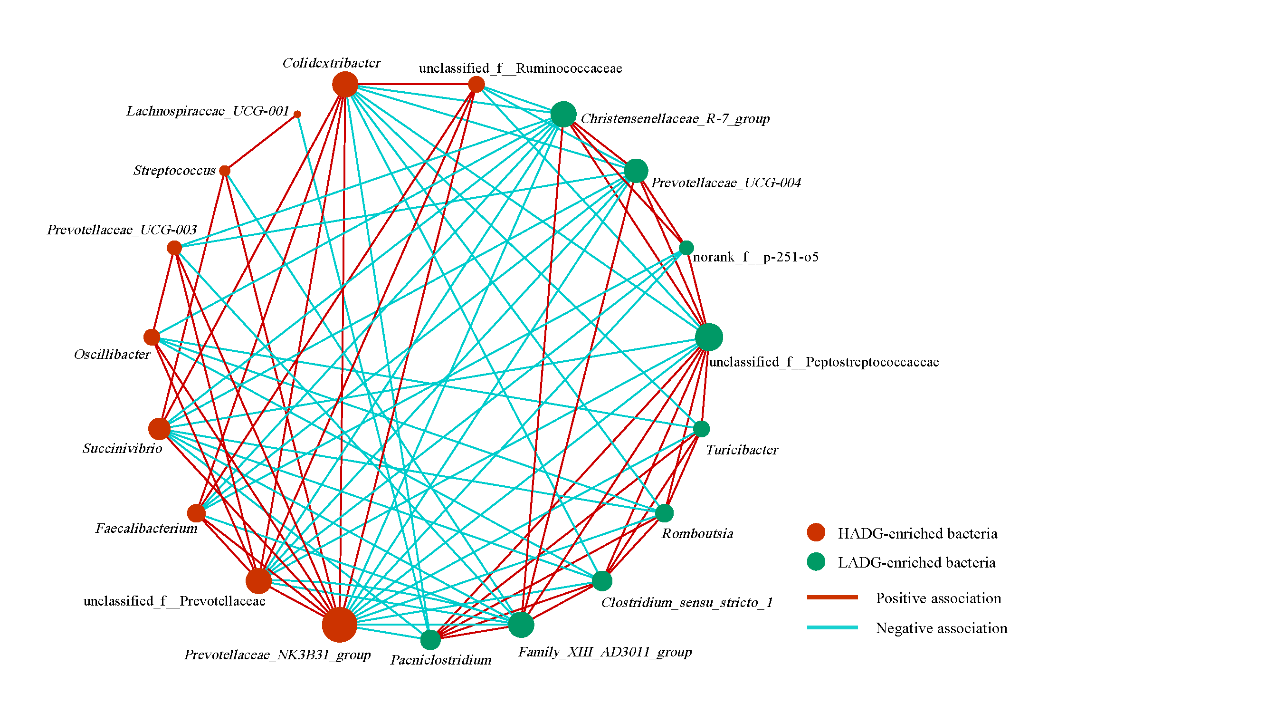


**Fig. S1** The relationship between ADG-related bacterial genera. Connections were detected based on Spearman’s rank correlations (*n* = 76, *P* < 0.05). Dot size represent number of connections with other taxa. Dot color represents the relationship between the relative abundance of bacterial genera and ADG. Edge color represents either positive or negative associations between bacteria


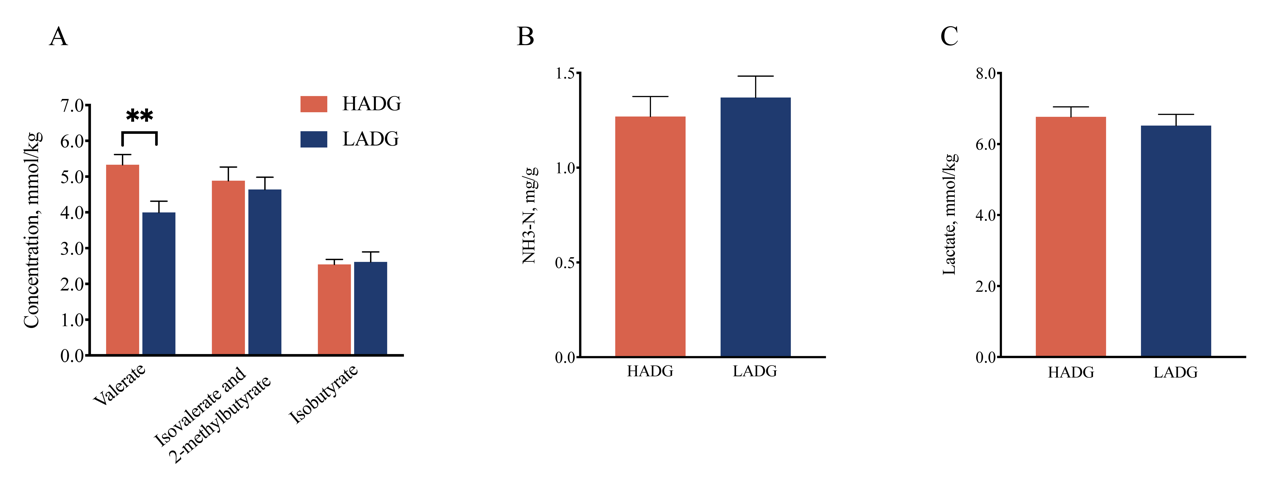


**Fig. S2** The difference in rectum minor SCFA, NH_3_-N and lactate between HADG and LADG group (6-month-old, *n* = 10 each group). Differences in data were assessed by student t test, The bars represent mean ± SEM. ^**^*P* < 0.01

**Fig. S3** The difference in rectum microbiota diversity and structure between HAL and LAL group (16-month-old, *n* = 8 each group). Significant differences were tested by Wilcoxon rank-sum test. The bars represent mean ± SEM. HAL: adult HADG goats, LAL: adult LADG goats


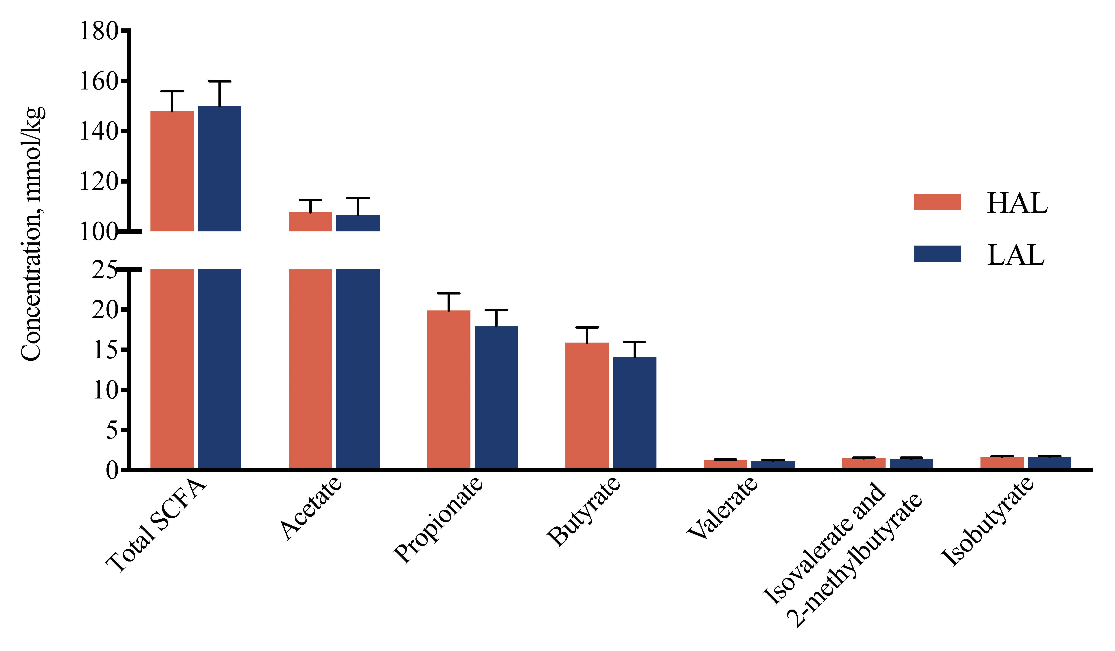


**Fig. S4** The difference in rectum SCFA between HAL and LAL group (16-month-old, *n* = 8 each group). Significant differences were tested by *t* test. The bars represent mean ± SEM. HAL: adult HADG goats, LAL: adult LADG goats


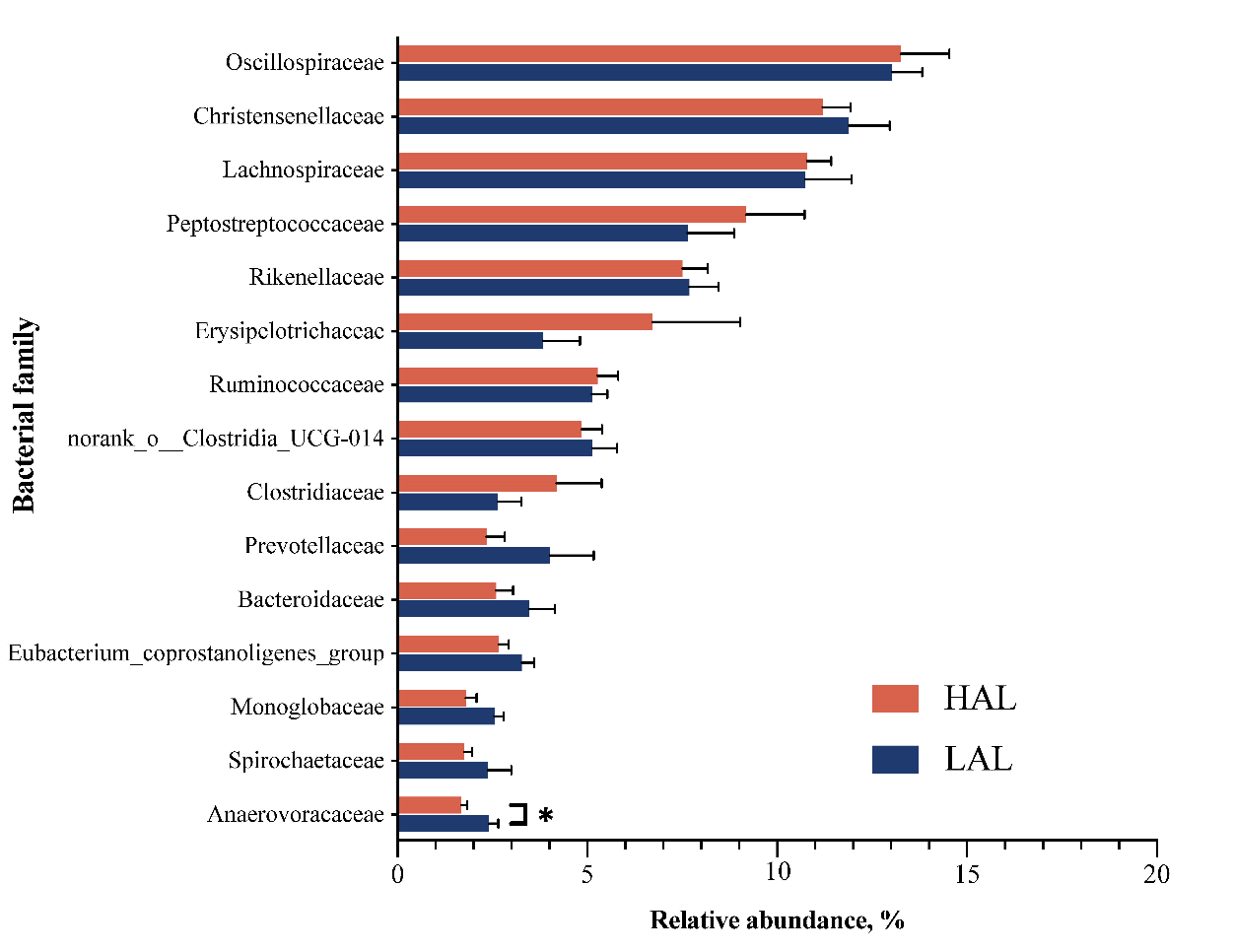


**Fig. S5** The difference in 15 most abundant rectum bacterial family between HAL and LAL group (19-month-old, *n* = 8 each group). Significant differences were tested by Wilcoxon rank-sum test. The bars represent mean ± SEM. ^*^*P* < 0.05. HAL: adult HADG goats, LAL: adult LADG goats


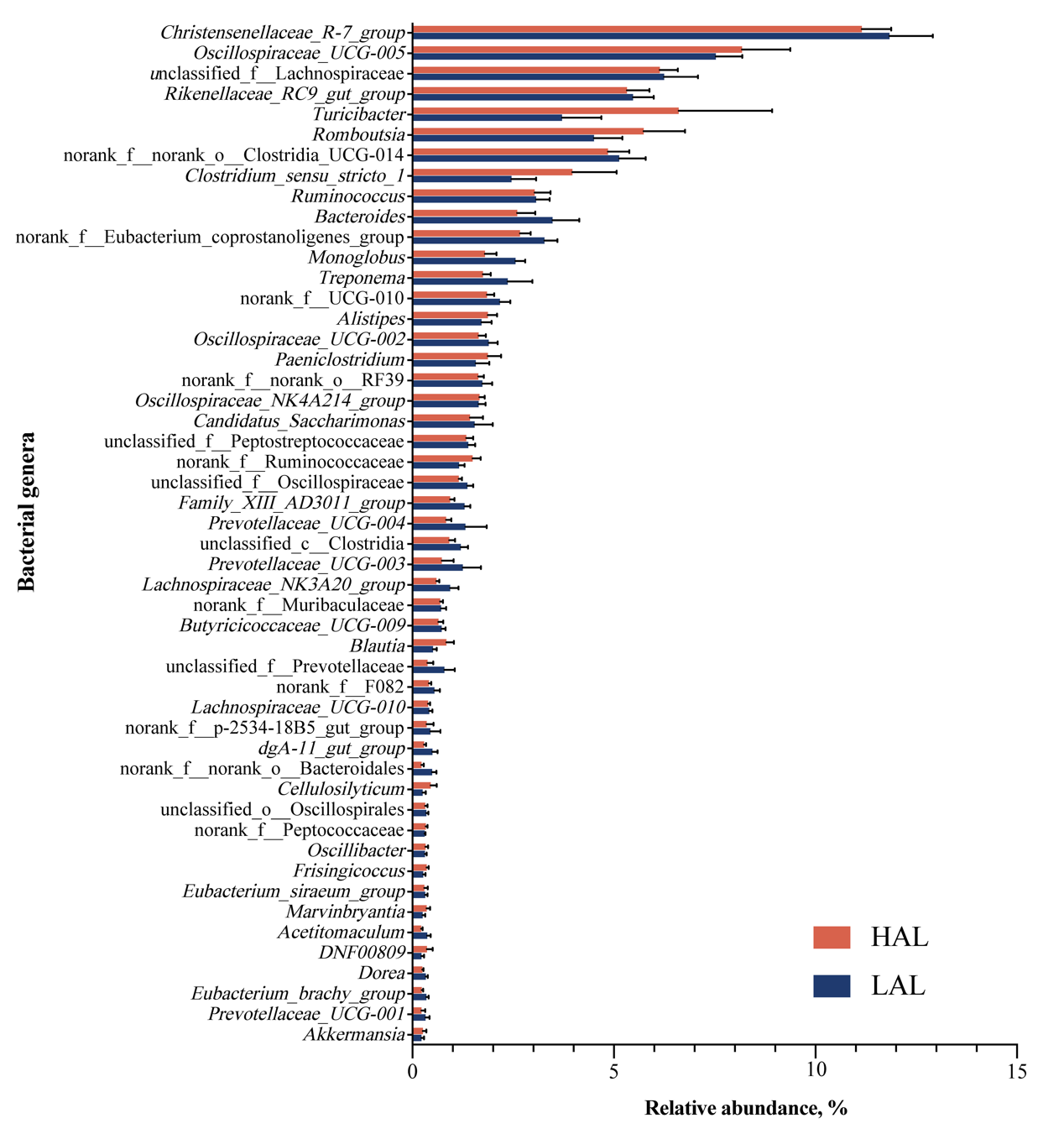


**Fig. S6** The difference in 50 most abundant hindgut bacterial genera between HAL and LAL group (19-month-old, *n* = 8 each group). Significant differences were tested by Wilcoxon rank-sum test. The bars represent mean ± SEM. HAL: adult HADG goats, LAL: adult LADG goats
